# The Therapeutic Potential of Amphetamine-like Psychostimulants

**DOI:** 10.3390/life13112180

**Published:** 2023-11-08

**Authors:** Bruno Pires, Luana M. Rosendo, Ana Teresa Brinca, Ana Y. Simão, Mário Barroso, Tiago Rosado, Eugenia Gallardo

**Affiliations:** 1 Centro de Investigação em Ciências da Saúde, Universidade da Beira Interior (CICS-UBI), 6201-506 Covilhã, Portugal; bruno.mg.pires@gmail.com (B.P.); luanamay.rosendo@hotmail.com (L.M.R.); anabrinca99@gmail.com (A.T.B.); anaaysa95@gmail.com (A.Y.S.); 2 Laboratório de Fármaco-Toxicologia, UBIMedical, Universidade da Beira Interior, 6200-000 Covilhã, Portugal; 3 Serviço de Química e Toxicologia Forenses, Instituto Nacional de Medicina Legal e Ciências Forenses, Delegação do Sul, 1150-219 Lisboa, Portugal; mario.j.barroso@inmlcf.mj.pt; 4 Centro Académico Clínico das Beiras (CACB)—Missão de Problemas Relacionados com Toxicofilias, 6200-000 Covilhã, Portugal

**Keywords:** amphetamines, stimulants, therapeutic

## Abstract

This review delves into the therapeutic applications of amphetamine-type stimulants such as lisdexamphetamine dimesylate, mixed amphetamine salts, 3,4-methylenedioxymethamphetamine (MDMA), dextroamphetamine, and phentermine. These compounds have been investigated for their potential in treating a range of psychiatric disorders, including attention deficit hyperactivity disorder (ADHD), drug dependence, post-traumatic stress disorder (PTSD), and obesity. Lisdexamphetamine dimesylate has shown promise in effectively treating ADHD symptoms in both children and adults. Additionally, it has been explored as a potential treatment for drug dependency and withdrawal, demonstrating encouraging results. Mixed amphetamine salts have also exhibited efficacy in reducing ADHD symptoms in adults. Future research should explore their potential use in treating bipolar disorder and cocaine dependence, considering the associated risks and benefits. MDMA-assisted psychotherapy has emerged as an innovative approach to treating PTSD, leading to sustained reductions in symptoms and even promoting post-traumatic growth. Furthermore, it has shown promise in managing anxiety related to life-threatening illnesses. Dextroamphetamine and phentermine have demonstrated efficacy in treating cocaine and opioid dependence, ADHD, and obesity. However, careful consideration and monitoring by medical professionals are essential due to the potential risks and benefits associated with them. In conclusion, amphetamine-type stimulants present a promising avenue for therapeutic interventions in various psychiatric conditions. Nevertheless, further research is necessary to comprehensively understand their mechanisms of action, dosage requirements, and long-term effects in different patient populations.

## 1. Introduction

Nowadays, psychotherapies are promoted as effective and cost-efficient treatments for a range of psychiatric disorders. Global awareness of psychiatric disorders has increased, with these disorders being associated with significant disease burdens and high rates of morbidity and mortality [1]. They often co-occur on a large scale with additional healthcare issues, aggravating the overall medical symptoms and complicating the management of medical conditions. As a consequence, there is a greater emphasis on the need for evidence-based pharmaceutical and psychotherapy treatments for psychiatric illnesses [2]. Pharmacotherapy is a type of psychotherapy that involves the use of medication in cases of substance misuse or addiction. It usually entails the use of typically abused drugs to aid therapy processes and promote recovery [3]. The administration of illicit drugs in therapy is an intricate and controversial issue. While most abused drugs present harmful effects, alongside being illegal, some of their compounds have shown potential therapeutic benefits when administered under controlled conditions [4].

Several drugs of abuse have been investigated for therapeutic purposes and shown to lead to significant and lasting positive changes in the overall status of patients. Psychedelic substances such as 3,4-methylenedioxyethylmethamphetamine (MDMA), psilocybin [5], and lysergic acid diethylamide (LSD) [6] have been studied in conjunction with therapy for various mental health conditions due to their properties [5,6]. MDMA is a stimulant and empathogen with the potential to treat post-traumatic stress disorder (PTSD), with its use involving limited administrations [7,8]. Psilocybin, a natural psychedelic compound found in certain species of mushrooms, has been investigated for its potential in the treatment of mental health conditions related to anxiety, depression, cancer, and substance use disorders [9,10]. Likewise, several studies have explored LSD’s therapeutic benefits for substance use disorders, PTSD, anxiety, and depression. These benefits include long-lasting mood improvements, symptom reduction, and increased well-being [6]. Dissociative anesthetics, such as ketamine, have also been studied and used to treat conditions such as suicidal ideation and severe depression [11]. This substance has shown rapid antidepressant effects, particularly in individuals who have not responded to other treatments [12].

Cannabis, a well-known recreative drug, has been legalized for medical use in several jurisdictions, being prescribed to alleviate symptoms associated with chronic pain, terminal cancer, nausea induced by chemotherapy, multiple sclerosis, and epilepsy [13]. Medical cannabis typically contains low levels of tetrahydrocannabinol (THC) and high levels of cannabidiol (CBD). THC is the psychoactive compound associated with the recreational use of marijuana [14].

Opioids have also been included in several clinical trials. In the treatment of opioid addiction, Methadone, a long-acting opioid agonist, is often combined with buprenorphine, a partial opioid agonist [15], or naltrexone, an opioid antagonist [16,17]. These medications help relieve withdrawal symptoms and reduce drug and alcohol cravings. Buprenorphine, in particular, reduces the risk of respiratory depression and overdose due to its ceiling effect, allowing individuals to stabilize their lives and engage in other aspects of treatment [15]. Naltrexone, on the other hand, neutralizes the pleasurable effects of opioids in the brain. Low-dose naltrexone has gained popularity due to its efficacy in managing chronic pain conditions [16,17]. It is critical to note that the therapeutic use of these substances requires the careful consideration of their potential risks and benefits [4]. Thoughtful deliberation, qualified guidance, and conformity to legal and ethical norms are mandatory prerequisites, especially since the toxicity mechanisms of all these drugs have not yet been fully established [18]. The substances’ characteristics, duration of treatment, and respective dosages need to be adjusted to the individual’s specific needs and continuously monitored by a qualified medical professional [4].

This subject is rapidly attracting interest, with ongoing research and debates about the safety, efficacy, and proper usage of chemical substances. It remains unknown how illicit drugs will be utilized in the near future, although novel responses, the clinical repurposing of old drugs, and the production of new compounds are inevitable outcomes. According to the United Nations Office on Drugs and Crime (UNODC), in 2019, approximately 27 million people worldwide were consuming amphetamines, with ages ranging between 15 and 64 years old [19]. Additionally, the 2021 report from the European Monitoring Centre for Drugs and Drug Addiction (EMCDDA) estimates that 1.4 million young adults (15–34 years old) have already consumed amphetamines [20], making amphetamine Europe’s second most consumed stimulant after cocaine [19].

The purpose of this review is to compile the most recent findings in the field of psychotherapy with amphetamine-type stimulants and analyze the structures, toxicities, metabolic pathways of detoxification, and clinical applications of these compounds.

## 2. Brief History

Natural amphetamines have been used for centuries through the consumption of various amphetamines-producing plants belonging to the genus *Ephedra* (family *Ephedraceae*), including *Ephedra sinica* [21]. *Ephedra* alkaloids are plant-derived alkaloids from the ancient Chinese medicine ma huang (*Ephedra sinica*), and ephedrine, one of its active alkaloids, is chemically similar to amphetamine [22]. Ephedrine, first isolated in 1885, proved to be effective in the treatment of asthma, making it a valuable medicinal ingredient due to its anti-asthmatic properties and oral administration. Pharmaceutical industries developed amphetamine as a synthetic substitute for ephedrine due to its limited active ingredient in the genus *Ephedra* [23].

Amphetamine, first synthesized in 1887, was available without prescription as a decongestant inhalant under the brand name Benzedrine from the 1930s to the 1960s [22,24].

Benzedrine was initially commercialized for asthma and narcolepsy but was later introduced for amphetamine-induced behavioral problems in children in 1937 [22,23]. Bradley’s research [23] revealed that a week’s treatment with amphetamine improved school performance for children with the disorder now recognized as attention deficit hyperactivity disorder (ADHD) [23].

Several athletes consumed benzedrine during the 1936 Olympics, which led to the initiative of creating methamphetamine under the commercial name Pervitin [23]. As a result, methamphetamine, a crystallized form of amphetamine, was first synthesized in 1938. This stimulant was widely distributed during World War II to assist pilots by alleviating the burdensome nature of long missions, warding off insomnia and hunger, increasing aggressiveness, and decreasing fatigue [22,23]. Moreover, in the 1950s and 1960s, it was widely prescribed as a medication for depression and obesity [25].

Amphetamine’s historical use has also been linked to the treatment of obesity, with amphetamine being used as an anorexic drug [22,23]. Its use in this context began in 1937, when it was discovered that the administration of amphetamine for the treatment of depression caused weight loss. However, it was only later that a reduction in food intake was recognized as a mechanism for weight loss [23].

In 1912, with the goal of synthesizing drugs and searching for vasoconstrictive agents, MDMA was created [25,26]. In the 1970s and 1980s, psychiatrists believed that this medication improved patient–doctor interactions and helped patients comprehend their issues. Nevertheless, it was added to the Schedule I drug list in 1985 and has remained there ever since [26]. The MDMA metabolite 3,4-methylenedioxyamphetamine (MDA), also known as the “love drug”, was briefly investigated for its potential medical applications, but it was never used for therapeutic purposes [27,28]. However, reports in the literature indicate that it was used as a tranquillizer in 1960 and as an appetite suppressant [25]. Several studies have suggested that this compound has potential if used as an adjunct to psychotherapy for depression [27,28].

In the 1950s, methylphenidate was discovered to be a potent stimulant. This drug was used to treat children with disorders such as attention deficit disorder (ADD) or ADHD [24].

Until the 1960s, experts excluded the possibility of amphetamine-like drugs being abused. Only in 1959 did the Food and Drug Administration (FDA) prohibit the sale of benzedrine inhalers without a prescription. However, groups of people encouraged the use of amphetamine pills, recommending them for a variety of physical and mental conditions. Amphetamine-like drugs are acknowledged in the scientific literature as having a high risk of neuropsychiatric side effects [23].

Amphetamine and related stimulants, such as methamphetamine, methylphenidate, phentermine and similar derivatives, hallucinogenic methoxy derivates (frequently referred to as “ecstasy”), and their natural congener ephedrine, are widely consumed nowadays [29]. Their recreational use is due to the fact that their effects include increasing energy and endurance, reducing appetite, boosting alertness, and enhancing confidence [22]. On the other hand, amphetamine derivatives are also used for therapeutic purposes (e.g., in the treatment of ADHD and Binge Eating Disorder (BED), the eating disorder most frequently associated with obesity [23]). For example, methylphenidate is still a first-line treatment for ADHD in children, and despite having reinforcing and discriminative stimuli, it remains effective. Lisdexamphetamine dimesylate, an oral prodrug of dextroamphetamine, is another amphetamine-like medication used to treat this disorder, and it is recommended when the response to methylphenidate or atomoxetine is inadequate [23].

## 3. Toxicokinetics

**Figure 1:**
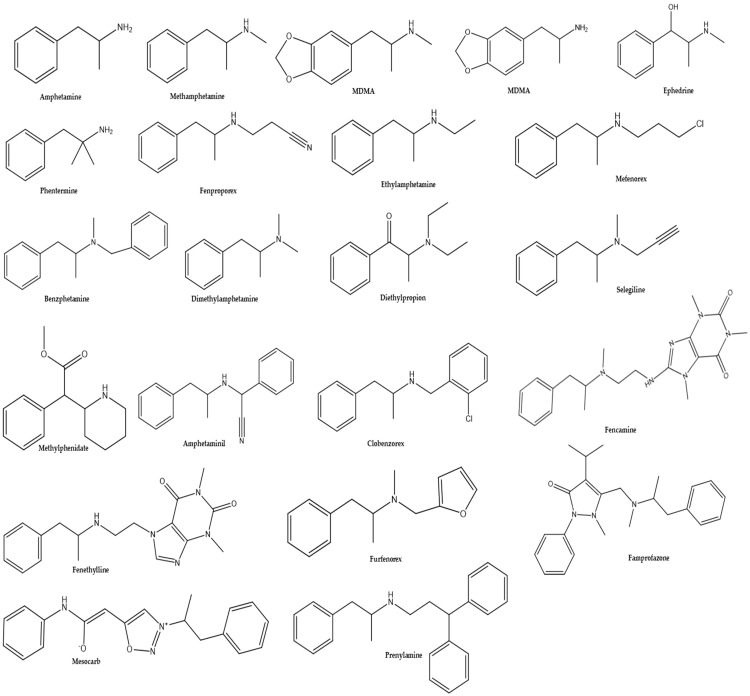
Chemical structures of amphetamine and amphetamine derivatives.

The basic chemical structure of amphetamine is composed of a phenethylamine backbone with a methyl group attached to the alpha carbon. Substitutions produce related compounds such as methamphetamine and MDMA, among others (Figure 1) [24,30].

Amphetamines are characterized by their low molecular weight and classification as chemically weak, basic drugs, with pKa values approximately around 9.9. They constitute a highly homogeneous class of drugs, exhibiting high oral bioavailability, a large volume of distribution, and minimal plasma protein binding (less than 20%). Elimination occurs via both renal and hepatic pathways, with an elimination half-life ranging from 6 to 12 h [21].

Typically appearing as a white, odorless, crystalline powder, amphetamines can be taken orally or by inhalation, either as a racemic mixture (levoamphetamine and dextroamphetamine) or as dextroamphetamine alone [22,31]. When administered orally, 90% of the dosage is absorbed in the gastrointestinal tract. The rate and extent of absorption of levoamphetamine and dextroamphetamine are not significantly different [21,31]. The onset of action for amphetamine occurs 30 to 45 min after consumption, influenced by factors such as dose, purity, or concurrent food intake [31]. Substances promoting acidification of the gastrointestinal tract decrease amphetamine absorption, while gastrointestinal alkalinization may increase it [31]. Peak plasma concentrations of amphetamine are typically reached after 4 h of ingestion, depending on the dose consumed. The time to achieve maximum concentrations ranges between 2 and 4 h [21,31]. Some studies have suggested that the consumption of high-fat foods may prolong the time needed to reach maximum concentrations, while other studies have shown that the consumption of amphetamine after a meal will result in maximum concentrations between 1 and 2 h, with simultaneous consumption having no effect on the time required to achieved maximum concentrations [31]. Less commonly used administration methods include intramuscular or intravenous injections, leading to maximum plasma concentration after 30 min. Other routes of administration include smoking and vaginal or anal insertion, but these administration routes are less frequently utilized [21,31].

As mentioned, amphetamines have poor protein binding capabilities, ranging from 16% to 20%, resulting in high bioavailability and easy diffusion from plasma to extravascular compartments. As a result, they tend to accumulate in perfused organs such as the liver, kidney, and lungs [31]. Furthermore, due to their lipophilic nature, they can easily traverse the blood–brain barrier and accumulate in the central nervous system [31]. There are no differences in the volumes of distribution or plasma binding between the two enantiomers of amphetamine, and while both are capable of reaching the central nervous system, dextroamphetamine tends to accumulate in larger quantities [21,31].

Amphetamine is metabolized via two principal oxidative pathways: oxidative deamination and hydroxylation [21,31]. Amphetamine undergoes *N*-deamination to be catalyzed by CYP450 isoenzymes, followed by the oxidation of the benzoic acid derivatives. These derivatives are then conjugated with glycine to form hippuric acid. Amphetamine can also be aromatically hydroxylated by CYP2D6, resulting in the active metabolite 4-hydroxyamphetamine [21,31]. Dextroamphetamine is metabolized faster than the equivalent enantiomer due to the stereoselectivity of amphetamine metabolism, resulting in half-lives that differ by 2 to 3 h. Dextroamphetamine has a half-life of 9 to 11 h, while levoamphetamine has a half-life of 11 to 14 h. As a result, disproportionate amounts of these two chemicals are observed during urine excretion, which is the primary route of elimination [21,31].

Methamphetamine, known by various street names such as speed, crank, meth, ice, or crystal, can be administered orally, intravenously, via snorting, via vapor inhalation, or via smoking methamphetamine hydrochloride salt. The less common route of administration method is oral ingestion [21,24]. Regardless of the route of administration, the terminal plasma half-life of methamphetamine is approximately 10 h, and acute effects may last up to 8 h following a single moderate dose. Smoked methamphetamine has a rapid onset of action like that of intravenous injection. The maximum concentration, opposed to the minimum of 3 h required after oral administration, is typically reached between 1h and 2.5 h. Additionally, the bioavailability of methamphetamine is significantly higher when smoked compared to when consumed orally (up to 90 vs. 67%, respectively). However, when snorted, its bioavailability only reaches about 79%, with peak plasma concentrations occurring after 4 h [21]. Methamphetamine’s distribution is similar to amphetamine and does not appear to be significantly affected by the route or timing of administration. Methamphetamine has been found to accumulate in drug users’ saliva, hair, and nails. According to these findings, methamphetamine accumulates in a number of organs, particularly the liver and lungs and, to a lesser extent, in the brain and kidneys [21].

MDMA, commonly known as ecstasy, “E”, “X”, “XTC”, and “ADAM”, is typically ingested orally, though it can also be snorted or administered rectally [24]. Initial effects are noted after roughly 30 min (ranging from 20 to 60 min), with the peak of the effects occurring between 60 and 90 min after ingestion [21]. After consumption, the pharmacological effects start to wear off 1h to 2h later, and basal levels are achieved over the next 4–6 h [21]. The concentrations of MDMA that accumulate in various tissues and organs are significantly higher than those found in plasma. This can be up to 18 times higher in the liver and 30 times higher in the brain [21].

CYP2D6 is also in charge of the metabolism of derivatives like methamphetamine, with the primary metabolic reaction being the *N*-demethylation of methamphetamine to amphetamine. This isoenzyme also catalyzes the 4-hydroxylation of methamphetamine’s aromatic ring, resulting in 4-hydroxymethamphetamine. β-oxidation follows the *N*-demethylation, producing norephedrine. Methamphetamine metabolizes less thoroughly than methylenedioxyamphetamine derivatives like MDMA, resulting in substantially higher levels of unaltered methamphetamine. However, these metabolites do not seem to contribute to the clinical effects of methamphetamine, as they are formed at low levels [21].

MDMA metabolism involves two main pathways: the first process involves the opening of the methylenedioxy ring, methylation of hydroxyl groups, and conjugation with glucuronide or sulfate; the second involves *N*-dealkylation into MDA, which retains its biological activity. Deamination and side-chain oxidation produce phenylketones, which are then conjugated with glycine and eliminated as hippuric acid [21]. Cytochrome P450 isoenzymes catalyze MDMA metabolic breakdown by demethylating the methylenedioxy ring and converting it to MDA. Demethylation occurs in vitro via biphasic Michaelis–Menten kinetics with high-affinity and low-affinity components [21].

Because renal excretion is the predominant elimination channel for amphetamines, urine pH has a large influence on their plasma half-life. Since these medications are weak bases, their half-life decreases with alkalization and increases with acidification. Due to the heterogeneity in elimination half-life, drug users take bicarbonate to extend the effects of the medication. The half-life is independent of the mode of administration and tends to be longer in amphetamine and methamphetamine addicts [21].

Within a pH range of 5 to 8, the proportion of administered amphetamine excreted in urine without biotransformation can range between 3 and 55.5%. Approximately 70% of a methamphetamine dose is eliminated within 24 h, along with small quantities of its metabolites, 4-hydroxymethamphetamine (15%) and amphetamine (10%). Methamphetamine has a half-life of 25 h and can build in urine when used regularly. The fraction of unmodified methamphetamine excreted in the urine decreases with increasing doses due to a reduced renal elimination rate or an increase in non-renal elimination rates. Regarding amphetamines with methylenedioxy substitution in the aromatic ring, such as MDMA, less amphetamine is excreted in urine (without biotransformation). The bulk of an MDMA dose is eliminated within the first 24 h of intake, with an elimination half-life ranging from 6 to 9 h. Approximately 80% of MDMA is excreted unchanged in urine after hepatic metabolism, with the remaining 20% excreted unchanged [21,24]. MDA urinary excretion represents less than 10% of the ingested dose of MDMA [21].

As mentioned previously, amphetamine is a stimulant and a euphoric, anorectic psychoactive substance [19] characterized by its complex and diverse pharmacological effects. Its primary mechanism of action relies on the release of postsynaptic catecholamines—dopamine and norepinephrine—from presynaptic terminals, leading to neuronal stimulation [21]. Therefore, addiction to amphetamines is linked to their actions on dopaminergic activity in the mesolimbic system. Amphetamine increases extracellular levels of monoamines like dopamine, noradrenaline, and serotonin by binding to monoamine transporters. Due to their similar molecular structure, amphetamines can enter presynaptic neurons via dopamine transporters and diffuse through the neural membrane. Once inside, they force dopamine molecules out of storage vesicles and expel them into the synaptic gap, causing the dopamine transporters to work in reverse. Additionally, amphetamines act as monoamine oxidase inhibitors, reducing dopamine reuptake [22,32].

Amphetamines derivatives such as amphetaminil, benzphetamine, clobenzorex, diethylpropion, dimethylamphetamine, ethylamphetamine, framproazone, fencamine, fenethylline, fenproporex, furfenorex, mefenorex, methylphenidate, mesocarb, prenylamine, phentermine, and selegiline are therapeutically used as sympathomimetics, anorectics, non-opioid analgesics, antiparkinsonian agents, or vasodilators [32,33]. It is crucial to distinguish between these derivatives when using these drugs therapeutically or recreationally. Drugs metabolized to methamphetamine and/or amphetamine present significant concerns in the interpretation of amphetamine-positive drug testing results. The presence of the parent compound or unique metabolite can be strong evidence for the involvement of the drug rather than amphetamine abuse [32,34].

Amphetaminil (α-methylphenethyl)amino]phenylacetonitrile) was developed in the 1970s and used for the treatment of obesity, ADHD, and narcolepsy [35,36]. This substance is decomposed in the organism to amphetamine, hydrocyanic acid, and benzaldehyde [36].

Benzphetamine (*N*-benzyl-*N*,-dimethylphenethylamine), a substituted sympathomimetic amine, contains a large *N*-benzyl that reduces excitatory qualities in the central nervous system (CNS) while keeping its anorexigenic capabilities. Benzphetamine, to a lesser extent, stimulates norepinephrine and dopamine. It causes a decrease in appetite by releasing dopamine from storage sites in the lateral hypothalamic feeding area. It is metabolized into dextroamphetamine and dextrometamphetamine, undergoing in vivo conversion to substances with high addiction and abuse potential [37]. 1-(4-hydroxyphenyl)-2-(*N*-benzylamino)propane is the main metabolite produced, and its identification in urine samples is unique and characteristic of the use of benzphetamine [36].

Clobenzorex (*N*-[2-chlorobenzyl]-amphetamine), an anorectic drug, is a precursor drug that is metabolized to amphetamine by the body and excreted in the urine. Because it is metabolized to amphetamine, the use of clobenzorex for therapeutic purposes may lead to the suspicion of amphetamine abuse. Drugs metabolized to methamphetamine and/or amphetamine present significant concerns when interpreting amphetamine-positive drug testing results.

Diethylpropion, also known as amfepramone, is a CNS stimulant with action similar to amphetamine that is clinically available as an anorectic agent [30,38]. It is widely used in South America for weight control and as a stimulant [30]. Diethylpropion is readily absorbed from the gastrointestinal tract and then metabolized to ethcathinone and *N*,*N*-diethylnorephedrine, excreted almost exclusively via the renal pathway. Only 2–4% of the drug is excreted unchanged. In human’s acidic urine conditions, about 30% of the dose is metabolized by deamination, followed by oxidation and conjugation to give hippuric acid [30,38].

Dimethylamphetamine is unavailable as a prescription drug and has no advantage over the use of methamphetamine due to fewer pharmacological effects [36].

Ethylamphetamine is used as an anorectic and to aid in the analysis of amphetamine-like drug metabolism processes. It is eliminated in its entirety and converted to amphetamine and 4-hydroxyethylamphetamine. The presence of the parent drug and an even larger concentration of the hydroxylated metabolite makes determining the usage of ethylamphetamine quite simple [36].

Framprofazone is a component of the multi-ingredient medication Gewodin, which presents antipyretic, analgesic, and sympathomimetic properties. The identification of the 3-hydroxymethylpyrazolone metabolite was determined to be definitive proof of famprofazone administration [36].

Fencamine is reported to be a CNS stimulant, and it has been used to treat depression [36].

Fenethylline, a CNS stimulant, is used for the treatment of ADD, narcolepsy, and depression. It is metabolized via two pathways. The existence of theophylline metabolites in combination with racemic amphetamine can lead to different interpretations, necessitating further discussions to confirm fenethylline consumption [36].

Fenproporex is the world’s second most popular amphetamine-like anorectic medication [30,36], and its derivatives are used in the treatment of male-type obesity. It was prohibited by the FDA due to a lack of efficacy and safety data. Despite this, the substance is extensively used, and its abuse can have serious economic and legal ramifications [30]. Fenproporex is degraded by one- and two-fold aromatic hydroxylation, followed by methylation and side-chain degradation by *N*-dealkylation to amphetamine via two partially overlapping processes. Amphetamine is converted to noradrenaline via beta-hydroxylation. Hydroxylation reactions are most likely responsible for the differences in amphetamine levels between people with a limited or extensive CYP2D6 metabolizer phenotype [10].

Furfenorex is an anorectic used to treat obesity. Among the many metabolites identified, 1-phenyl-2-(*N*-methyl-*N*-valerolactonylamino)propane was discovered to be unique in its properties and application, establishing furfenorex as the source of amphetamine and methamphetamine [36].

Mefenorex is a sympathomimetic agent with properties similar to those of amphetamine but with less effects on the cardiovascular system. It is widely used as an anorectic adjunct in the short-term treatment of moderate to severe obesity. The presence of the chloropropyl side chain protects mefenorex from *N*-dealkylation, and aromatic hydroxylation is the predominant metabolic pathway [36].

Methylphenidate is a cyclized amphetamine derivative with two chiral centers. This phenethylamine was synthesized in 1944 and later patented in 1954. Methylphenidate is promptly and completely absorbed from the gastrointestinal tract after oral ingestion. Peak concentrations occur 1 to 2 h after administration of the dosage. Methylphenidate’s pharmacokinetic half-life is generally 2 h, ranging from 2 to 7 h. De-esterification converts methylphenidate to ritalinic acid. The psychostimulant treats ADHD by releasing dopamine and blocking the dopamine transporter, limiting synaptic cleft dopamine reuptake and raising extracellular dopamine [30].

Mesocarb is a stimulant that presents fewer side effects compared to amphetamine. It has been used to counteract the effects of benzodiazepines and to increase workload capacity and cardiovascular function [36].

Prenylamine is used to treat angina pectoris due to its properties as a coronary vasodilator since it works as a calcium channel blocker. It causes a dose-dependent decrease in anaphylactic histamine release, like amphetamine and methamphetamine. Amphetamine, 4-hydroxyamphetamine, norephedrine, 4-hydroxynorephedrine, and diphenylpropylamine were identified as metabolites [36].

Selegiline is used in the treatment of Parkinson’s disease. Additionally, it might display some antidepressant and psychotropic effects. The metabolism of deprenyl involves biotransformation into desmethyldeprenyl, R-(−)-methamphetamine, R-(−)-amphetamine, and their P-hydroxy derivatives [36].

Phentermine is a sympathomimetic amphetamine stimulant with a methyl substituent on the phenylethylamine side chain, which lowers CNS activation [37].

Currently, no reliable lab tests can predict ADHD. Its symptoms are usually associated with disruptions in the genes that control specific dopamine transporters (DRD5, DRD2, DRD4) [39]. Furthermore, structural changes in the prefrontal cortex, corpus striatum, cerebellum, and white matter pathways have been implicated in ADHD’s pathophysiology [39]. The dopamine and norepinephrine systems have been proposed as the underlying neurobiological mechanisms that lead to ADHD [39,40]. Dopamine transporter deficiencies (DAT) impair attention, while changes in dopamine receptor responses contribute to delayed brain maturation in individuals with ADHD [39]. Dopamine dysfunction also affects executive functions, learning, and overall inattention [40]. Medications like methylphenidate (a stimulant related to amphetamines) target ADHD by blocking dopamine and norepinephrine reuptake transporters, alleviating ADHD symptoms [40]. Cholinergic neurotransmission impairments also contribute to cognitive deficits in ADHD, with catecholaminergic and cholinergic neuromodulation dysfunction playing roles ADHD symptomatology [40].

When it comes to PTSD, modifications in gut microbiota have also been shown to enhance vulnerability to trauma by promoting the production of specific metabolites that influence various brain functions and systems [41]. Experiments conducted in mice by S. Laudani et al. [41] demonstrated that susceptible mice exhibited an increased relative abundance of pro-inflammatory bacteria such as *Ruminococcaceae* and *Lachnospiraceae*. These bacteria are known to produce toxic metabolites, including ammonium, indole, and P-cresol, through the degradation and fermentation of proteins [41].

P-cresol can hinder myelination by obstructing myelin gene expression and differentiation [41]. Furthermore, P-cresol can act as an inhibitor of dopamine beta-hydroxylase, affecting the dopaminergic system, with a single intravenous administration of P-cresol elevating the dopamine metabolism [41]. In this context, it is important to note that dopaminergic dysfunctions play a central role in the pathophysiological mechanisms underlying PTSD [41]. An abnormal dopamine level in the prefrontal cortex may contribute to the disruption of the top-down inhibitory control exerted by this region over the limbic regions, a key factor responsible for the occurrence of both intrusion and hyperarousal symptoms [41].

Moreover, there was a substantial up-regulation of the gene responsible for encoding the dopamine D3 receptor in the prefrontal cortex of susceptible mice in [41]. This up-regulation can also contribute to the failure of the top-down inhibitory control exerted by the prefrontal cortex over the limbic regions [41]. Notably, both genetic and pharmacological blockades of D3Rs have been linked to the facilitation of the prefrontal cortex’s top-down control over subcortical brain regions [41]. It is plausible that the detrimental impact of P-cresol on myelination may be connected to the abnormal stimulation of D3Rs in the prefrontal cortex via dopamine [41].

Given the crucial role of dopamine in the prefrontal cortex (PFC) for working memory, both in animals and humans, Westphal et al. [42] conducted a study to investigate whether working memory performance could be enhanced in individuals with PTSD by administering tolcapone, a medication that inhibits the degradation of dopamine by catechol-O-methyl transferase (COMT), thus increasing cortical dopamine levels. By highlighting the significance of cortical dopamine in working memory function, this study provided additional evidence that boosting cortical dopamine levels can result in improved cognitive control in individuals with more pronounced PTSD symptoms [42].

Ross and Bockstaele [43] also emphasize the critical role of catecholaminergic systems in PTSD. The modulatory neurotransmitters dopamine and norepinephrine determine which memories are salient, commanding attention and influencing behavioral outcomes [43]. Dopamine and norepinephrine modify the neurochemical environment to aid emotional memory regulation through dopaminergic and adrenergic receptors [43].

Internal and external reward reinforcement is driven by dopamine’s activation of D1-like receptors, while signaling via D2 receptors is known to mediate conditioned responses to fear-inducing stimuli [43]. The locus coeruleus–norepinephrine (LC-NE) system also plays a pivotal role in the central stress response, coordinating cognitive responses to potential threats by scanning the environment [43].

As previously mentioned, a leading hypothesis regarding circuit dysfunction in PTSD suggests a failure of top-down cortical inhibitory neurons to suppress the reactivation of trauma-related memory traces, leading to difficulty in inhibiting the limbic system [43]. Numerous studies have demonstrated the significant involvement of catecholamine neurotransmitters and the pathophysiology of PTSD.

## 4. Therapeutic Applications

In this section, we review recent studies involving amphetamine-like substances in the therapeutic treatment of psychiatric disorders, including attention deficit hyperactivity disorder (ADHD), drug dependence, post-traumatic stress disorder (PTSD), and obesity. A PubMed search was performed using the following keywords: “Amphetamines” AND “Therapy”. The search was restricted in terms of publication date so that only publications from the last 3 years were retrieved, meaning that all selected articles were published between 2020 and 2023. Additional filters included the following: Books and Documents, Case Reports, Clinical Study, Clinical Trial, Clinical Trial Phase I, Clinical Trial Phase II, Clinical Trial Phase III, Clinical Trial Phase IV, Controlled Clinical Trial, Randomized Controlled Trial, and Humans.

### 4.1. Lisdexamphetamine Dimesylate

Several studies have shown the safety and efficacy of using lisdexamphetamine dimesylate (LDX) in the treatment of several medical conditions. ADHD and drug dependency are examples of conditions wherein this compound has shown promising results in terms of recovery and therapeutic applications. Regarding ADHD, five articles published in the last 3 years have investigated the use of LDX to treat the symptoms related to this condition (Table 1). Childress et al. [44,45,46] published three studies that highlighted the safety, tolerability, and efficacy of LDX in children aged 4–5 years [44,45,46]. All of the studies had a similar structure, encompassing screening and washout, dose optimization, dose maintenance, and a safety follow-up, with doses ranging from 10 to 30 mg LDX per participant [44,45,46]. Focusing on a different age range, Adler et al. [47] demonstrated that for concentrations between 30 and 70 mg a day, the used of LDX in adults with ADHD and Comorbid Sluggish Cognitive Tempo (SCT) was beneficial, showing significant improvements in SCT when compared to a placebo [47]. Wang et al. [48] adopted a different approach, studying striatal and thalamic functional connectivity (FC) using static (time-averaged) and dynamic (time-varying) measures to then correlate those results with ADHD symptom improvements after treatment with a long-acting LDX [48]. Despite limitations due to the small sample size and wide age range, the results support that the active treatment with LDX significantly improved ADHD-related symptoms [48]. The most frequently reported treatment-emergent adverse events (TEAEs) across the five studies were decreased appetite, insomnia, irritability, dry mouth, and headaches, all of which were considered to be mild to moderate in severity [44,45,46,47,48].

LDX has also demonstrated a beneficial effect with respect to treating illicit drug dependency and withdrawal. Since there is no medication approved for methamphetamine use and withdrawal, LDX has the potential to be used as an agonist for therapy aimed at ameliorating withdrawal symptoms [49]. Acheson et al. [50] used a tampered amount of LDX to study the feasibility and safety of its use in treating acute methamphetamine withdrawal [50]. The regimen started at 250 mg daily, with a 50 mg reduction per day until reaching 50 mg on day 5, and the results demonstrated that the regimen applied was a safe and feasible treatment [50]. On the other hand, LDX has also been tested in methamphetamine-dependent adults, with doses similar to those of Acheson et al. [50] showing very promising results [51]. A reduction in methamphetamine usage and cravings was observed, while the treatment was also confirmed to be safe, with adverse events being reportedly mild to moderate in severity [51]. This use of LDX also applies to other drugs of abuse, such as cocaine. Mariani et al. [52] tested this application in the context of cocaine use disorder (CUD) to determine the safety, tolerability, and optimal dosing of LDX, reaching the conclusion that an application of approximately 140 mg is safe and generally well tolerated, claiming it to be appropriate for future studies [52].

Due to the characteristics of amphetamines and LDX, it is only natural to explore their potential in the context of eating disorders/obesity cases. An interesting case report by Preddy et al. [53] relates the case of a young boy with Prader–Willi syndrome (PWS) who used LDX to manage weight loss and obesity [53]. PWS is associated with significant weight gain and obesity, and this symptom has been unsuccessfully controlled in patients for many years [53]. For this particular patient, LDX showed an immediate and significant reduction in appetite and consequent weight loss. A significant improvement in functional status and overall day to day living came as a result of this treatment, and the patient remained on LDX [53].

### 4.2. Mixed Amphetamine Salts

ADHD affects a great part of the world population, continuously prompting the testing and creation of treatment alternatives to LDX. Psychostimulants are commonly employed in ADHD treatment, and mixed amphetamine salts (MAS) are another type of amphetamine derivatives that have shown promising results when used for this purpose (Table 1). Fick et al. [54] and Adler et al. [55] tested the long term safety and efficacy of a triple-bead mixed amphetamine salt in adults with ages ranging from 18 to 55 years [54,55]. Fick et al. [54] observed that doses of 25–75 mg of MAS significantly reduced ADHD symptoms in adults, although no dose–response efficacy was statistically proven [54]. Long term safety was assessed by Adler et al. [55] in a 12-month exposure study that confirmed the safety and efficacy of MAS, with the most commonly reported TEAEs being similar to that of LDX (insomnia, headache, dry mouth, upper respiratory tract infection, decreased appetite, weight decreased, and nasopharyngitis [55]).

Each patient is different, and each treatment with MAS may induce different results and TEAEs. Sedation and lethargy are extremely rare adverse effects that may appear with the administration of psychostimulants, and it has been shown that MAS are no exception [56]. Thus, it is of extreme importance for clinicians to always follow up on MAS administration and therapy, especially when it comes to treating ADHD symptoms in patients on the autism spectrum [56].

ADHD can be accompanied by other conditions, such as bipolar disorder (BD) or symptoms indicative of both disorders occurring simultaneously [57]. Existing findings suggest that psychostimulants can effectively and safely manage the manic symptoms of BD when used without a mood stabilizer. However, these medications carry an elevated risk of inducing psychotic and manic symptoms, especially in the absence of a mood stabilizer [57]. Further research into the use of MAS for treating BD without relying on mood stabilizers should be undertaken while remaining mindful of the potential risks associated with BD and its associated psychotic and manic symptoms [57].

CUD remains a significant public health issue, and amphetamines are among the few medications showing promising therapeutic results [58]. Impairments in dopamine transmission predict non-responsiveness to behavioral therapy, underscoring the potential significance of drugs like MAS, which have substantial control over the dopaminergic system. However, a number of medical, psychosocial, and practical factors, such as the possibility of abuse or diversion, health issues like cardiovascular disease, the worsening of psychiatric conditions like BD or anxiety disorders, and insurance coverage of off-label psychostimulants, make it difficult to treat cocaine or other stimulant use disorders with psychostimulants [58].

Combining MAS with topiramate has yielded promising results, indicating that individuals with cocaine dependence (those who consume cocaine at least nine times per month) have a higher likelihood of achieving abstinence when treated with a combination therapy involving extended-release MAS and topiramate compared to those receiving a placebo [59].

### 4.3. 3,4-Methylenedioxymethamphetamine (MDMA)

Studies have demonstrated that, when used in conjunction with psychotherapy, MDMA is effective in significantly reducing the clinical symptoms associated with PTSD [60]. However, this treatment has not been extensively studied in relation to posttraumatic growth (PTG), which encompasses positive transformations in self-perception, interpersonal relationships, or overall life philosophy [60]. To address this, Gorman et al. [60] evaluated the effects of MDMA-assisted psychotherapy on PTG and PTSD, demonstrating more significant reductions in the severity of PTSD symptoms compared to the placebo group. Importantly, these improvements were sustained, both immediately after treatment and at the 12-month follow-up. The findings of Jerome et al. [61] further support the potential of MDMA-assisted psychotherapy, indicating that this approach, involving two to three sessions with proper preparation and follow-up, holds promise for maintaining clinically significant improvements in PTSD symptoms for at least 1 to 3.8 years following treatment.

In a multi-site phase 3 clinical trial, Mitchell et al. [62] employed a randomized, double-blind, placebo-controlled approach to investigate the effectiveness and safety of MDMA-assisted therapy for individuals with severe PTSD. The study included participants with common comorbidities, such as dissociation, depression, a history of alcohol and substance abuse, and childhood trauma. Their findings reveal that MDMA-assisted therapy exhibits remarkable efficacy in treating severe PTSD and that it was well tolerated and safe, even for individuals with comorbid conditions [62]. Moreover, MDMA-assisted therapy for severe PTSD has the potential to result in subclinical improvements in alcohol use. Importantly, there was no evidence to suggest that MDMA increased the risk of illicit drug use [63]. Similar positive results were obtained concerning sleep regulation and fear conditioning and extinction [64,65]. The most frequent adverse events observed were elevated blood pressure, headache, anxiety, and tachycardia, but all reported adverse events were of mild to moderate severity, temporary in nature, and did not necessitate medication or intervention. In other words, no serious adverse events were observed [65].

Initial evidence suggesting that MDMA-assisted psychotherapy has the potential to be a safe and viable treatment for individuals with life-threatening illnesses (LTIs) in terms of reducing anxiety and alleviating other associated psychiatric symptoms has also been supported by Wolfson et al. [66]. The results of their study support the feasibility of utilizing MDMA-assisted psychotherapy as an innovative approach for the long-term management of anxiety related to LTIs [66].

### 4.4. Amphetamine, Phentermine, Dexamphetamine, and Dextroamphetamine

In a study conducted by Faraone et al. [67], the treatment effect size of amphetamine extended-release oral suspension (EROS) was assessed at various time points throughout the day (1, 2, 4, 6, 8, 10, 12, and 13 h post-dose). The findings indicated that amphetamine EROS demonstrated a strong and consistent effect in treating ADHD symptoms in children aged 6–12 years. Beneficial effects were observed to start early in the morning and persist throughout the entire day.

Swerdlow et al. [68] examined the effects of amphetamine on auditory frequency modulation learning (auditory learning) in targeted cognitive training for schizophrenia [68]. Amphetamine doses of 2.5–5 mg showed significant improvements in auditory learning, with maximal effects observed at 5 mg. These effects were consistent at 60 and 210 min after administration.

Cutler et al. [69] investigated the efficacy of dextroamphetamine transdermal system (d-ATS) as an alternative to oral formulations for ADHD treatment [69]. d-ATS proved effective in treating ADHD in children and adolescents, showing significant improvements and a large effect size and number needed to treat (NNT) of 2–3 for a clinically meaningful response [69]. The treatment demonstrated good tolerability, with minimal reports of dermal reactions and a low incidence of TEAEs overall [69].

Similar to MAS, dexamphetamine has displayed promising results in the treatment of cocaine dependence [70]. In a study by Blanken et al. [70], the efficacy of sustained-release dexamphetamine was demonstrated in reducing cocaine use among patients with cocaine dependence. This treatment not only led to decreased cocaine use but also showed potential for improving health-related outcomes in individuals with co-occurring heroin dependence who were undergoing heroin-assisted treatment [70]. In a case report by Palis et al. [71], dextroamphetamine was prescribed to a 51-year-old male with concurrent opioid and stimulant use disorder undergoing injectable opioid agonist treatment, resulting in a significant reduction in cocaine use [71].

Phentermine has been found to be an effective short-term treatment for obesity, according to research conducted by Márquez-Cruz et al. [72]. Their study revealed that the 30 mg dosage of phentermine was more effective than the 15 mg dosage after a 12-week follow-up, although there was no significant difference at the 24-week mark. Both long-term doses of phentermine were well tolerated and considered safe. Approximately 80% of the subjects experienced positive outcomes after continuing to use phentermine for an additional three months. In a study conducted by Pérez-Cruz et al. [73], the short-term administration of phentermine at a daily dose of 15 mg, combined with a lifestyle intervention program, resulted in a 19% reduction in hepatic steatosis and a greater loss of fat mass in kilograms among patients who were candidates for bariatric surgery. These findings suggest that phentermine could be a viable treatment option in preoperative interventions for individuals undergoing (or set to undergo) bariatric surgery.

**Table 1:** Amphetamine-like compounds used in studies published between 2020 and 2023 to test their therapeutic potential in the treatment of ADHD.

Amphetamine-like Compounds Used	Dosage (mg)	Study Conditions	Type of Study	Duration (Weeks/Months)	Participants (Ages)	Results	Reference
MAS	12.5 to 50 mg	4 weeks of dose optimization; 11 months of dose maintenance; and a 30-day (±5 days) follow-up period	Phase 3, multicentered; open-label extension of two Phase 3 studies	12 months	505 Adults (between 18 and 55 years of age)	Long-term safety and tolerability; long-term effectiveness in the treatment of ADHD symptoms for up to 12 months.	[55]
LDX	10; 15; 20;	Four periods: screening and washout; dose optimization (6 weeks); dose maintenance (2 weeks); and safety follow-up (1 week)	Phase 2, multicentered; open-label dose-optimization study	~11 months	Children (between 4 and 5 years of age)	Safety and tolerability were consistent with its known effects in older children and adolescents with ADHD; the titration scheme used was well tolerated and conferred treatment benefits.	[46]
MAS	25, 50, and 75	2-week screening phase; 1- to 4-week washout phase; 6-week forced-dose double-blind treatment phase; 30-day (±5 days) follow-up period	Randomized; placebo; controlled, double-blind, forced-dose study	7 months	Adults (between 18 and 55 years of age)	All triple-bead MAS doses assessed (25, 50, and 75 mg) were statistically superior to the placebo treatment in reducing ADHD symptoms in adults; there was no statistical evidence of a dose–response relationship for efficacy; the short-term safety and tolerability profiles of the triple-bead MAS were like other long-acting stimulants.	[54]
LDX	20; 30 and 70 mg/d.	10-week crossover trial with 2 double-blind treatment periods consisting of 4 weeks each and an intervening 2-week single-blind placebo washout	Randomized placebo; controlled, crossover trial	27 months	38 Adult (between 18 and 60 years of age)	Significant improvements after LDX vs. placebo were showed for comorbid sluggish cognitive tempo in adults with ADHD.	[47]
LDX	5, 10, 15, 20, or 30	long-term study including four periods: screening and washout; dose optimization; dose maintenance; safety follow-up	Phase 3, open-label; multicentered	52 weeks	113 children (between 4 and 5 years of age)	At doses between 5 and 30 mg/d, the treatment was found to be safe and well tolerated; no new safety signals were identified. The efficacy profile was consistent, with robust improvements in ADHD.	[44]
LDX	10-, 20-, and 30-mg doses	Screening and washout (1–4 weeks); fixed-dose titration (3 weeks); dose maintenance (3 weeks); safety follow-up (1 week)	Phase 3 randomized; double-blind; multicentered, parallel group; PBO-controlled; fixed-dose study	~13 months	Children (between 4 and 5 years of age)	LDX was generally well tolerated; no new safety signals were identified.	[45]
LDX	from 30 to 70 mg daily	N.A.	Randomized; placebo; controlled trial	12 weeks	58 children and young adults (between 6 and 25 years of age)	Active treatment significantly improved the inattentive, hyperactive/impulsive, and emotional lability subscales.	[48]
amphetamine extended-release oral suspension	2.5–20 mg/day	1–4 weeks of screening; 5-week open-label period	Phase 3, randomized; dose-optimized; double-blind, placebo; controlled; laboratory classroom assessment	10 weeks	99 children (between 6 and 12 years of age)	Robust and consistent effects beginning early in the morning and continuing throughout the day in the treatment of symptoms.	[67]
d-ATS	5, 10, 15, and, 20 mg; (equivalent to approved doses of 4.5 mg/9 h, 9 mg/9 h, 13.5 mg/9 h, and 18 mg/9 h, respectively	Open-label dose-optimization period. Randomized, crossover double-blind treatment period	Open-label dose-optimization period preceded a randomized, crossover double-blind treatment	7 weeks	110 children and adolescents (between 6 and 17 years of age)	d-ATS was effective in the treatment of ADHD in children and adolescents; d-ATS represents an important innovation for the known population of patients with ADHD who respond better to amphetaminethan methylphenidate.	[69]

## 5. Conclusions

Psychotherapies and evidence-based pharmaceutical treatments play crucial roles in addressing the global burden of psychiatric disorders. This review focused on the therapeutic applications of various amphetamine-type stimulants, such as lisdexamphetamine dimesylate, mixed amphetamine salts, MDMA, and other amphetamine derivatives, including dextroamphetamine and phentermine. The findings indicate that lisdexamphetamine dimesylate has shown promising results in treating ADHD in both children and adults, as well as in addressing drug dependency and withdrawal, making it a versatile treatment option.

Mixed amphetamine salts have also demonstrated efficacy in alleviating ADHD symptoms in adults, and further research is needed to explore their potential use in treating bipolar disorder and cocaine use disorder.

MDMA-assisted psychotherapy has emerged as a novel approach in the treatment of PTSD, and it has exhibited significant and sustained reductions in PTSD symptoms. Furthermore, it has shown promise in promoting post-traumatic growth and managing anxiety related to life-threatening illnesses.

Lastly, dextroamphetamine and phentermine have demonstrated efficacy in treating conditions such as cocaine and opioid dependence, ADHD, and obesity. These substances require careful consideration and monitoring by medical professionals due to their potential risks and benefits.

Overall, the therapeutic application of amphetamine-type stimulants continues to evolve, and further research is needed to fully understand their mechanisms of action, dosage requirements, and potential risks and benefits for various psychiatric disorders.

## Data Availability

Data sharing is not applicable to this article.

## Abbreviations

ADD	Attention deficit disorder
ADHD	Attention deficit hyperactivity disorder
Amphetaminil	α-methylphenethyl(amino)phenylacetonitrile
BD	Bipolar disorder
Benzphetamine	*N*-benzyl-N,-dimethylphenethylamine
CBD	Cannabidiol
Clobenzorex	*N*-(2-chlorobenzyl)-amphetamine
CNS	Central nervous system
COMT	Catechol-*O*-methyl transferase
CUD	Cocaine use disorder
CYP	Cytocrome P450
d-ATS	Dextroamphetamine transdermal system
DAT	Dopamine transporter deficiencies
EMCDDA	European Monitoring Centre for Drugs and Drug Addiction
EROS	Extended-release oral suspension
FC	Functional connectivity
FDA	Food and Drug Administration
LC-NE	Locus coeruleus–norepinephrine
LDX	Lisdexamphetamine Dimesylate
LSD	Lysergic acid diethylamide
LTI	Life-threatening illness
MAS	Mixed amphetamine salts
MDA	3,4-methylenedioxyamphetamine
MDMA	3,4-methylenedioxymethamphetamine
NNT	Number needed to treat
PFC	Prefrontal cortex
PTG	Posttraumatic growth
PTSD	Post-traumatic stress disorder
PWS	Prader–Willi syndrome
SCT	Sluggish cognitive tempo
TEAE	Treatment-emergent adverse events
THC	Tetrahydrocannabinol
UNODC	United Nations Office on Drugs and Crime
